# Spatial Visual Imagery (SVI)-Based Electroencephalograph Discrimination for Natural CAD Manipulation

**DOI:** 10.3390/s24030785

**Published:** 2024-01-25

**Authors:** Beining Cao, Hongwei Niu, Jia Hao, Xiaonan Yang, Zinian Ye

**Affiliations:** 1 School of Mechanical Engineering, Beijing Institute of Technology, Beijing 100081, China; 3120200302@bit.edu.cn (B.C.); nhwfend@bit.edu.cn (H.N.); yangxn@bit.edu.cn (X.Y.); 3220230339@bit.edu.cn (Z.Y.); 2 Yangtze Delta Region Academy, Beijing Institute of Technology, Jiaxing 314019, China; 3 Key Laboratory of Industry Knowledge & Data Fusion Technology and Application, Ministry of Industry and Information Technology, Beijing Institute of Technology, Beijing 100081, China

**Keywords:** natural CAD manipulation, EEG-based interaction, SVI, spatial EEG features, multi-feature fusion

## Abstract

With the increasing demand for natural interactions, people have realized that an intuitive Computer-Aided Design (CAD) interaction mode can reduce the complexity of CAD operation and improve the design experience. Although interaction modes like gaze and gesture are compatible with some complex CAD manipulations, they still require people to express their design intentions physically. The brain contains design intentions implicitly and controls the corresponding body parts that execute the task. Therefore, building an end-to-end channel between the brain and computer as an auxiliary mode for CAD manipulation will allow people to send design intentions mentally and make their interaction more intuitive. This work focuses on the 1-D translation scene and studies a spatial visual imagery (SVI) paradigm to provide theoretical support for building an electroencephalograph (EEG)-based brain–computer interface (BCI) for CAD manipulation. Based on the analysis of three spatial EEG features related to SVI (e.g., common spatial patterns, cross-correlation, and coherence), a multi-feature fusion-based discrimination model was built for SVI. The average accuracy of the intent discrimination of 10 subjects was 86%, and the highest accuracy was 93%. The method proposed was verified to be feasible for discriminating the intentions of CAD object translation with good classification performance. This work further proves the potential of BCI in natural CAD manipulation.

## 1. Introduction

Computer-Aided Design (CAD) is well-developed, and its functions are very complete. However, some CAD manipulations like translation, rotation, and zooming are still performed with conventional devices like a mouse, keyboard, and so on, which are not conducive to the natural expression of design intentions. At present, more and more intuitive interactive modes have been applied to CAD manipulation [1,2,3,4], enabling designers to interact with CAD more directly and naturally by distinguishing design intentions with physiological signals. Basically, the brain produces design intentions and sends messages to some corresponding parts like the hands and eyes, which eventually execute the task. However, people still need to express their minds physically. These interaction modes are still not intuitive enough. Thus, it is interesting to investigate whether a computer can obtain design commands from the brain directly.

It is well known that manipulation intentions, especially some singular ones, are directly related to certain brain activities [5,6]. Thus, it is probable that these manipulation commands can be output from the brain directly [7]. It has been verified that some simple manipulations like horizontal translation and clockwise and counterclockwise rotation can induce certain brain patterns [8,9]. Decoding commands from brain information could be a complementary method for natural interaction. Therefore, the first step is to investigate the feasibility of discriminating a manipulation intention from the brain directly before support system development and further evaluation of this strategy.

The brain–computer interface (BCI) has been increasingly applied in daily life as an intuitive interactive mode that outputs human intentions without the involvement of the peripheral nervous system or muscle tissue [10,11]. In the research community, the application of BCIs in desktop object controls like cursor movement and CAD manipulation is very promising [12,13]. Designers can communicate directly with the computer using their brains to realize the simple manipulation control of geometric objects in CAD.

Currently, steady-state visual evoked potential (SSVEP), P300, and motor imagery (MI) are widely used in manipulation control [14,15]. However, both SSVEP and P300 are external stimulation-induced-based EEG paradigms, which require users to focus on the corresponding stimulus elements constantly. Users are prone to visual fatigue when staring at the flashing interface. With regard to motor imagery [16,17], users are instructed to imagine the movement of their right hands, left hands, feet, and other parts of their bodies without executing the movement. Although motor imagery is an endogenous paradigm, it also faces cognitive pressure problems due to the fact that the imagination of limb motion itself is unrelated to CAD manipulation. Moreover, designing a product with CAD is a time-consuming job that requires designers to sit in front of computers for several hours. These mature BCI paradigms are prone to cause physical fatigue because designers have to focus on the visual stimulus or imagine the movement of limbs for hours. These behaviors have little relation to the design work, which wastes the designers’ energy in vain. Once designers spend a lot of time on these unrelated behaviors, they cannot focus on design work and express their innovative thinking well. Therefore, a more natural BCI paradigm is essential for CAD manipulation. 

Visual imagery (VI) is a mental imagery paradigm that only requires users to imagine the corresponding scene from a third-person perspective. In this paradigm, additional cognitive activity that is unrelated to the task is eliminated. VI is a better EEG-based interactive strategy for CAD manipulations. Designers only need to imagine the manipulation scenes to express their intentions. At present, studies on VI mainly focus on some special cases, such as specific image imagination [18], visual motor imagination [19], and so on. Obviously, these conventional VI paradigms are still not suitable for CAD manipulations due to their inappropriate imagination scenarios. Therefore, a VI paradigm that is suitable for CAD manipulation is needed. Recently, some VI paradigms related to spatial cognition have been proposed [20,21,22]. In these studies, participants were asked to imagine a scene where an object moves in a given direction. The spatial visual imagery (SVI) paradigm seems to be extremely fitting for some single CAD manipulations. Especially in CAD assembly tasks, where translation and rotation manipulation are needed, designers could output the commands by just imagining the spatial pattern, such as moving left/right or rotating clockwise/counterclockwise. In this way, designers could express their intentions directly from their brains without any extra behavior. However, the research into SVI is not complete. Previous studies have not clearly explained the discrimination mechanism of this paradigm. Thus, further research on SVI is extremely necessary. 

Recently, some studies have pointed out that the spatial imagery-like perception of motion is related to the dorsal stream that passes through the occipital, middle temporal, and parietal lobes [23,24]. It is not clear whether there are some distinctive spatial EEG patterns in the dorsal stream-related areas. Moreover, the feasibility of spatial feature-based SVI discrimination has not been verified yet. Therefore, we attempt to study a spatial feature-based discrimination strategy for SVI. Two SVI tasks, imagining objects moving left and imagining objects moving right, are designed. An SVI experiment is conducted to analyze the spatial features of EEG. Finally, a multi-input model containing spatial features and a deep learning algorithm is built for EEG recognition.

The rest of this paper is organized as follows. Section 2 introduces some work related to this study. Section 3 shows the details of the SVI experiments. Section 4 depicts the method for spatial feature extraction and gives the feature analysis. Section 5 presents the discrimination model and its performance. Finally, Section 6 concludes this paper and outlines our future work.

## 2. Related Work

### 2.1. Research on Spatial Visual Imagery (SVI) EEG

SVI is a suitable paradigm for CAD interaction where designers only need to imagine the corresponding manipulation scenes. In recent years, several studies have shown that people exhibit distinct patterns of brain activation when perceiving motions in different directions. Consequently, some discriminating methods for the SVI paradigm have been proposed.

Teresa Sousa et al. studied three classes of spatial visual imagery patterns [20]. The subjects in their experiment were asked to imagine a static dot, a dot with two opposing motions in the vertical axis, and a dot with four opposing motions in vertical and horizontal axes. They used the power spectral density (PSD) of six anterior electrodes as the features and obtained a classification accuracy of 87.64%. Yuki Seto et al. conducted a VI experiment where subjects were asked to imagine an arrow pointing in four directions (up, down, left, and right) [25]. Fast Fourier Transform (FFT) and principal component analysis (PCA) were used to extract features. A three-layer neural network was applied for classification. The study achieved its best result of 55% accuracy when data from the O1 electrode were selected. Kenta Tomonaga et al. conducted a similar experiment and used the same method to recognize different SVI tasks [26]. Notably, a better accuracy (above 60%) was achieved when the electrodes located in the occipital and parietal lobes were selected. K. Koizumi et al. proposed views on SVI from a high-frequency perspective [27]. Subjects were asked to imagine the movement of a drone in three planes (up/down, left/right, and forward/backward). Then, the PSD features in the gamma band were extracted, and a support vector machine (SVM) was used for classification. An accuracy of 84.6% was obtained when the prefrontal cortex electrodes were selected. However, participants were also asked to read silently in the experiment, which may have induced some EMG components.

Thomas Emmerling et al. applied fMRI to study the SVI paradigm [28]. Two groups of experiments were conducted in their study. One group of subjects imagined motion in the left, right, up, and down directions, and the other group imagined motion in the four diagonal directions. The average classification accuracy was around 50%. Only two subjects who imagined diagonal motion had results higher than 80%. The highest accuracy was achieved in the V3 and V4 regions, which are located in the occipital lobe. The authors concluded that V3 and V4 seem to be predominant areas for decoding the direction of motion during SVI. M. Serdar Bascil et al. conducted a 1-D SVI experiment, where subjects were required to imagine the horizontal motion of a cursor (moving left or moving right) [9]. Average signal power and power difference were applied to extract EEG features in the alpha band (8–12.5 Hz) and beta band (13–30 Hz) from 18 electrodes across all brain regions. Then, PCA was used to reduce the feature dimension to obtain the effective feature. Finally, LVQ, MLNN, and PNN were used for pattern recognition. The result illustrated that the alpha and beta power levels in the right occipital lobe were significantly higher when the subject imagined the left motion, and vice versa. Results of only two subjects were given in their studies. The average recognition accuracy was 93.05%. They also set a group for a 2-D experiment, where the subjects were required to imagine the motion of a cursor in four directions (up, down, left, and right) [29]. The PSD feature of EEG was extracted in the 2-D experiment. Then, PCA and ICA methods were applied to reduce the dimension of the PSD feature, and a 70-dimensional compressed feature was obtained. LS-SVM, LVQ, MLNN, and PNN were applied for classification. The average accuracy of the 2-D task was 89.83%. However, a significant EEG pattern was not observed in the 2-D experiment. Also, the feature extracted with PCA and ICA methods was less interpretable.

As mentioned above, spectrum features and dimensionality reduction algorithms have been widely applied in SVI studies. Only frequency domain information has been investigated in the previous studies. Although the PCA algorithm can be used to obtain more effective features, it is highly dependent on the characteristics of individual data. Thus, PCA is prone to overfitting in the case of insufficient data, resulting in poor transferability of the recognition model. Therefore, more effective features need to be explored. In general, no unified conclusion has been reached in the current studies on SVI in the community of EEG recognition.

### 2.2. Research on the Spatial Features of Imagery-Related EEG

As endogenous EEG paradigms, VI and MI can be collectively referred to as mental tasks. Event-related desynchronization (ERD) phenomenon occurs on the contralateral sensorimotor cortex [30], which has been proven to be a significant topological pattern of MI. According to the characteristics of ERD, some studies extracted the spatial features of MI EEG signals and achieved excellent classification [31,32,33]. Previous studies indicate that SVI is related to the dorsal stream, which can be seen as a special spatial pattern. Thus, these spatial features, which are fitting for MI classification, likely apply to SVI as well.

Common Spatial Pattern (CSP) is one of the most commonly used spatial feature extraction methods [34]. A set of spatial filters is obtained with the diagonalization of a matrix, which is applied to projecting the original EEG data into a common space where the variance difference between the two classes of projected data is maximized. A Filter Bank CSP (FBCSP) method was proposed by Kai Keng Ang et al. [35], which filters the original EEG into several frequency bands and calculates the CSP feature of each band. Then, some discriminative features are extracted with feature selection methods. Rongrong Fu et al. proposed a sparse CSP method [36]. Sparse processing and iterative search models are used in CSP to eliminate the influence of irrelevant electrodes. Neethu Robinson et al. combined CSP with wavelet [37]. High-resolution decomposition of EEG and high-temporal location can be realized with the Wavelet-CSP method.

Functional connectivity (FC) is another kind of effective spatial feature of EEG signals. The phase lag index (PLI) was calculated as the functional connectivity value, and an accuracy of 94% was obtained in the previous work [38]. Yijun Wang et al. calculated the phase locking value (PLV) of EEG signals collected in the primary motor area (M1) (local scale) and SMA (large scale) as functional connectivity features. They achieved a left/right-hand imagery classification with an accuracy of 87.02% [39]. In the community of signal processing, high coherence between two EEG signals implies high cooperation and synchronization between underlying brain regions within a certain frequency band [40]. Coherence has been selected as the FC index in many cases of EEG recognition. Fali Li et al. applied coherence-based FC to show the different electrode linkage patterns of various MIs [41]. Correlations between different electrodes are also used as the FC feature. Linear correlation has been used for MI classification [42]. However, correlation FC is prone to be affected by the volume conduction effect [43], which may result in poor performance.

In the research community of EEG, few people used spatial features to interpret the SVI paradigm. It remains to be verified whether the spatial features of SVI EEG are recognizable and how to realize the pattern recognition with these features.

## 3. Experiment

### 3.1. Purpose of the Experiment

The poor naturalness of the conventional CAD interaction mode causes a high design load, which impacts the innovative thinking of CAD users badly.

Assembly is one of the most important CAD tasks, in which the designed parts are fitted together with manipulations like translation, rotation, and scale [44]. The scenes of objects moving tend to appear in designers’ minds when they want to output CAD assembly commands like translation. During this period, some visuospatial information is processed by the brain and may induce some special brain activities related to SVI. Thus, some assembly commands may be obtained by recognizing SVI EEG directly.

**Figure 1:**
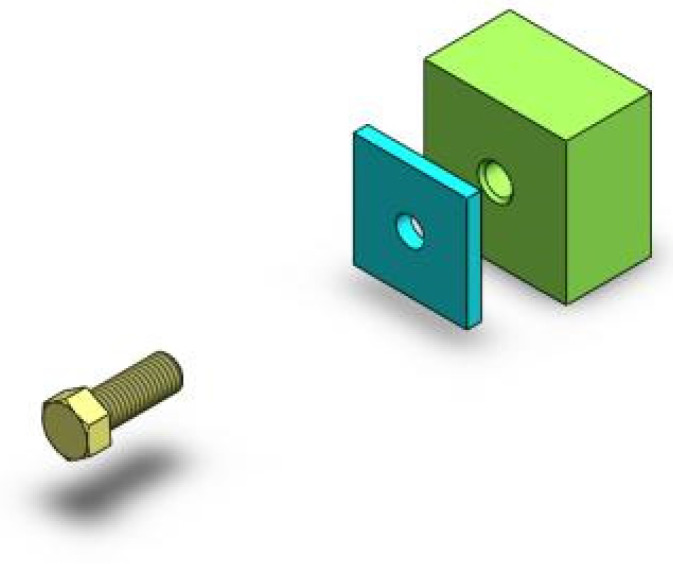
A screw assembly scene in the CAD environment.

As mentioned above, SVI may be a good solution that designers can use to intuitively express manipulation intention. To study a suitable SVI paradigm for CAD manipulation and verify the feasibility of single-trial discrimination, an experiment for SVI EEG collection was conducted in this work. Considering that translation manipulation is commonly seen in assembly tasks, a “screw assembly” operation task was set as the experiment scene, as this task mainly includes translation work. In this way, SVI EEG induced by real-world CAD manipulation can be obtained. In this experiment, subjects were asked to imagine a screw moving toward a hole part in the cue direction. The assembly scene is shown in Figure 1. 

### 3.2. Experiment Details

#### 3.2.1. Experimental Protocol

**Figure 2:**
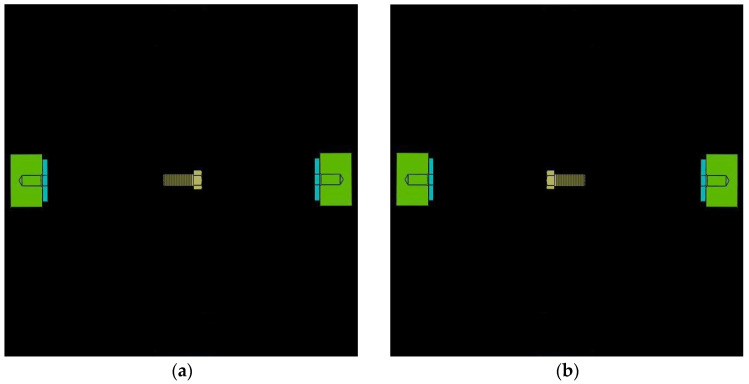
Experiment stimulus: (**a**) left motion stimulus and (**b**) right motion stimulus.

In this experiment, a moving stimulus was used. The first reason is that the controlled object is moving continuously when a user gives the control commands in the practical application. The second reason is that most subjects claimed that they could obtain better visual imaginations with moving stimuli in the preliminary experiment. The stimulus interface is shown in Figure 2, where the screw is initially located at the central initial position. Figure 2a is the left motion stimulus and Figure 2b is the right motion stimulus.

The motion velocity of the screw was set to 100 pixels per second. Subjects were given 4500 ms for visual imaging in each trial. The distance between the screw and the object part was adjusted to 450 pixels. Thus, the screw could just reach the object part at the end of the stimulus. 

**Figure 3:**
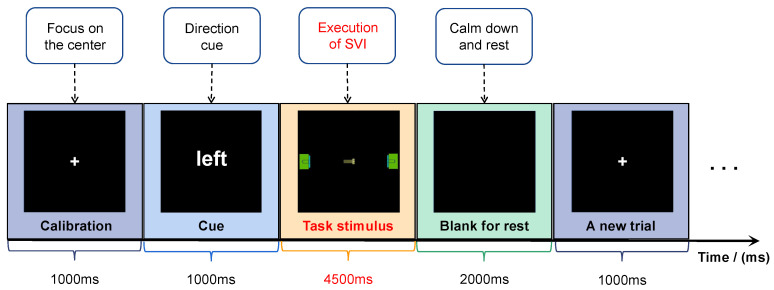
Schedule diagram of the experiment.

A schedule diagram of the experiment is shown in Figure 3. At the beginning of each trial, a calibration interface with a central cross appears for 1000 ms. The subjects are required to focus on the center position. Then, a semantic cue appears for 1000 ms to indicate the motion direction of the screw in the trial. The stimulus duration is 4500 ms. The subjects are supposed to execute SVI tasks with their eyes fixed at the center of the screen. To eliminate artifact influence, the subjects are not allowed to move their bodies when they are imagining. The left task and right task are given randomly. At the end of each trial, a blank interface appears for 2000 ms, allowing the subjects to rest their eyes. One trial of the experiment lasts for 7500 ms. Eight rounds of experiments are conducted for each subject. Each round contains 20 trials, including ten left and ten right motion imagination tasks. A total of 160 samples, comprising 80 right SVI tasks and 80 left SVI tasks, are collected.

#### 3.2.2. Implementation of the Experiment

The experiment paradigm procedure was implemented with E-Prime in this work. The stimuli interface was made with Psychtoolbox 3.0. A cube screen with a resolution of 1280 × 1024 was used to present the paradigm to the subjects. For the acquisition module, a 64-electrodes Neuroscan electroencephalograph (including a pair of mastoid reference electrodes) and a Synamps2 amplifier (Compumedics Neuroscan, Charlotte, NC, USA) were used to collect the EEG signals. 

The EEG signals were converted into digital signals and stored in a computer with CURRY 8 software. In this work, the sampling rate was set to 500 Hz. The left and right mastoid electrodes were selected as the reference electrodes. The vertical EOG and horizontal EOG were monitored to eliminate the artifacts caused by eye movements. To slice the data epoch, special marks were set for the stimuli and stored with the EEG signals together. E-prime was able to send the TTL marks to CURRY 8 via a parallel interface line when stimuli were presented.

#### 3.2.3. Subjects and Environment

Referring to the subject number in the previous studies [45,46], ten subjects, including 7 males and 3 females, participated in this experiment. They ranged in age from 21 to 26. All subjects were mentally healthy and had normal vision or corrected-to-normal vision. They filled out a Vividness of Visual Imagery Questionnaire (VVIQ) [47] before formal experiments to evaluate the clearness of the imagination they could achieve. Considering all subjects are Chinese, the VVIQ was translated into Chinese correctly. The VVIQ scores are given in Table 1. The full score of the VVIQ is 80. Here, all the subjects scored above 48 (past 60%), which indicated that they were able to imagine a scene relatively clearly. Additionally, all subjects were asked to write down what kinds of SVI they had executed actually in the experiment, and the details of their imaginations are shown in Table 2. It is normal for different subjects to have different strategies.

Before starting the experiment, each subject completed a practice task to ensure that they could achieve a clear imagination.

#### 3.2.4. Data Acquisition and Preprocessing

Original EEG signals were collected with the amplifier and then converted into digital data. Data and mark numbers were received and stored simultaneously.

The original data were re-referred with CURRY 8 first. Here, the left and right mastoid electrodes were selected as the reference. To eliminate the EOG, EMG, and other artifacts, ICA was used to make blind signal separation with EEGLAB [48] and EEG was divided into several independent components. Then, these significant artifact components were removed. With the assistance of mark reference, the data epochs were obtained by slicing the EEG with a 4500 ms time window. Thus, each epoch contained 2250 data frames.

## 4. Feature Extraction of SVI

**Figure 4:**
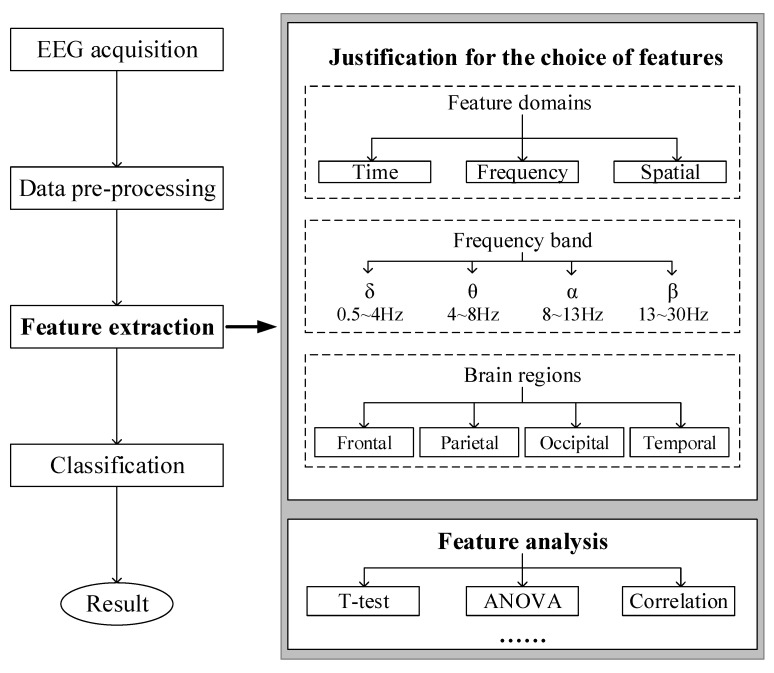
Flow chart of feature extraction.

EEG data were collected in the experiment and required further processing to realize recognition. The procedure of EEG recognition is shown in the left part of Figure 4. Particularly, feature extraction is an essential session because effective EEG features can not only enhance the performance of the classification model but also reveal some significant patterns associated with the corresponding brain activities. The right part of Figure 4 shows the procedure of feature extraction. Firstly, the selection of features is determined based on three aspects. Drawing on previous knowledge, it is necessary to determine which feature domains, frequency bands, and brain regions are used to extract features. Secondly, the extracted features are analyzed with statistical methods to verify their significance. Finally, these features are used to build a classification model.

### 4.1. Spatial Feature Extraction for SVI

#### 4.1.1. A Conduction Pathway-Based Hypothesis for Feature Extraction

In the research community of visual cortices, two typical conduction theories called “ventral pathway (VP)” and “dorsal pathway (DP)” have been proposed. Both VP and DP originate in the occipital lobes to obtain visual information. But these two paths terminate in different regions, determining their different functions. VP terminates in the temporal lobe, while DP terminates in the parietal lobe. Previous studies indicate that VP is mainly relative to object recognition [49], color perception [50], and so on. DP is mainly activated in cases like spatial information processing [51], direction perception [52], and so on. 

DP originates in the early visual cortex V1/V2. Then, it arrives at motion area V5/MT, which is located at the intersection of the occipital lobe, parietal lobe, and temporal lobe. The V5/MT area is mainly responsible for processing complex visual motion stimuli. Finally, DP terminates in the inferior parietal area, which participates selectively in the processing of visuospatial information [51]. 

In the SVI task, subjects were asked to imagine a screw moving in a fixed direction and try to project this virtual scene on the screen. Previous work has pointed out that some regions like MT of DP are extremely associated with the sense of direction. In the SVI task of this work, subjects perceived the direction of motion (left and right). Therefore, the lobes located at DP may be recruited in SVI tasks. Although the vision pathway mechanism has been shown in many studies, it remains unclear whether the EEG features extracted in the visuospatial perception regions in two SVI tasks are considerably different and whether these features can be used for EEG discrimination. Therefore, a hypothesis that there may be some evident differences between the EEG features in the DP area in different SVI tasks is proposed in this work.

Among these regions related to DP, V5/MT and the parietal lobe with visuospatial information processing abilities may be crucial parts of SVI EEG processing. Because DP involves the cooperation of several brain regions, it is necessary to investigate the relationship of EEG collected with different electrodes. Particularly, spatial EEG features are mostly used to represent EEG relationships between different regions. Among the existing spatial features, CSP is a method used to enhance the difference in multi-electrode data distribution between two classes by mapping data with a matrix called a CSP filter. Functional connectivity values like cross-correlation and coherence can indicate the relationship between different electrodes. Therefore, CSP, cross-correlation, and coherence are selected to verify our hypothesis with feature analysis.

Furthermore, it is necessary to discard redundant data before feature extraction. Considering that SVI-related EEG mainly exists in the occipital and parietal lobes, data from these two positions were selected as the input of feature extraction. Twenty-eight electrodes including TP7, TP8, CP1–CP6, CPZ, P1–P8, PZ, PO3–PO8, POz, O1, O2, and Oz were selected for feature extraction. To find the band of interest, the original EEG was filtered into δ (0.75–4.5 Hz), θ (4–8 Hz), α (8–12.5 Hz), and β (12–28 Hz) and processed with the proposed method, respectively. An FIR digital band-pass filter with a bandwidth of 3 dB was applied for filtering. Previous research indicated that visual imagery may be related to the alpha band EEG [53,54], so EEG signals in the alpha band were selected for data analysis of our work.

#### 4.1.2. CSP Features of SVI

CSP is a data-driven EEG feature extraction method that can be divided into two parts. The first part is training the CSP filter with EEG data from different SVI tasks. In this part, covariance matrices of the different classes of EEG are calculated first. Then, these covariance matrices are joint diagonalized and whitened to obtain a mapping matrix, commonly referred to as a CSP filter or spatial filter. In the second part, different classes of EEG signals are projected into a common space where the sum of the eigenvalues of different classes of projected EEG is 1. Thus, the variance difference in the projected data is enhanced. The application condition of CSP is that the distribution of EEG signals collected from several electrodes should be significantly different in various imagination tasks, which implies that the EEG topographic patterns are supposed to be distinctive in different tasks.

The calculation process of the CSP filter is referred to in this work. The CSP features are calculated with the following formulas.

(1) $$Z_{1}=WE_{1}$$

(2) $$Z_{2}=WE_{2}$$

(3) $$f_{1}=\log(1+\mathrm{var}(Z_{1}))$$

(4) $$f_{2}=\log(1+\mathrm{var}(Z_{2}))$$

where *E*_1_ and *E*_2_ denote two EEG signals of different electrodes. *W* is the CSP filter. Here, the original trained CSP filter is in the shape of 28 × 28 because 28 electrodes are selected. According to the previous work, CSP features obtained with the first few lines and the last few lines of CSP filters are distinctive [55], and the number of selected CSP filters typically ranges from 2~6 [56]. Thus, only the first three rows and the last three rows are selected in this work after offline testing, as these six filters showed the best performance. Therefore, the final shape of *W* is 6 × 28. *Z*_1_ and *Z*_2_ are projected data in the common space. Finally, the variances of Z_1_ and Z_2_ are calculated and converted into CSP features logarithmically (*f*_1_ and *f*_2_). 

#### 4.1.3. Cross-Correlation (CC)-Based FC Features of SVI

During the visual imagination process, EEG signals are always induced and transported to other related regions, which form a specific signal stream in the brain. Thus, there may be some time lags between the EEG signals of various electrodes. Cross-correlation is a frequently used method in the temporal analysis of two potentially related signals, which gives a function for time lag and relational degree. When the EEG stream goes through two electrodes sequentially, the signals of these two electrodes may exhibit strong cross-correlation. Considering that conductive patterns may vary in different SVI tasks, cross-correlation is applied in this work. To build an FC network, the peak value of the function is selected as the connectivity value [57]. The scope of the time lag is set to 4 s, which is high enough to observe the conductive relation of every electrode couple. The formula is given as follows, where *x*(*t*) and *y*(*t*) are two signals and *f_c_* is the connectivity value.

(5) $$fc=\max(\int_{-\infty }^{+\infty }x(t)y(t+\tau )dt),\tau \in [-4,4]$$

#### 4.1.4. Coherence-Based FC Features of SVI

The coherence value of each pair of selected channels is calculated with Formula (6). Then, a brain network is constructed with the coherence connectivity. The phase lag can be evaluated with coherence. A high coherence indicates that the phase lag between two signals is relatively constant and the connection between these two signals is relatively strong. Thus, the coherence value is an effective index to investigate the phase correlation of SVI EEG signals.

(6) $$C_{xy}(f)=\frac{|P_{xy}(f){|}^{2}}{P_{xx}(f)P_{yy}(f)}$$

where *x*(*t*) and *y*(*t*) denote the EEG signals of two different channels. *P_xy_*(*f*) is the cross-spectral density of *x*(*t*) and *y*(*t*) at frequency *f*. *P_xx_*(*f*) and *P_yy_*(*f*) are the auto-spectral densities of *x*(*t*) and *y*(*t*). *C_xy_*(*f*) is the coherence result of *x*(*t*) and *y*(*t*) at frequency *f*. Coherence values of 8, 9, 10, 11, and 12 Hz are calculated, respectively, and then their average value is selected as the final connectivity index. Finally, a 28 × 28 coherence matrix is obtained.

### 4.2. Analysis of Spatial Features for SVI

Three spatial features including CSP features, coherence FC, and cross-correlation FC, are used in this work. The analysis is combined with conduction pathway theory to determine if these features have significant differences in various SVI cases.

#### 4.2.1. Analysis of CSP Features

One CSP feature consists of six feature values in this work. A *t*-test (α = 0.05) is used to analyze if these feature values are significantly different between the two tasks. The result is shown in Table 3. Sixty values were tested, and forty-four (73.3%) of them are significantly different between the two tasks.

**Figure 5:**
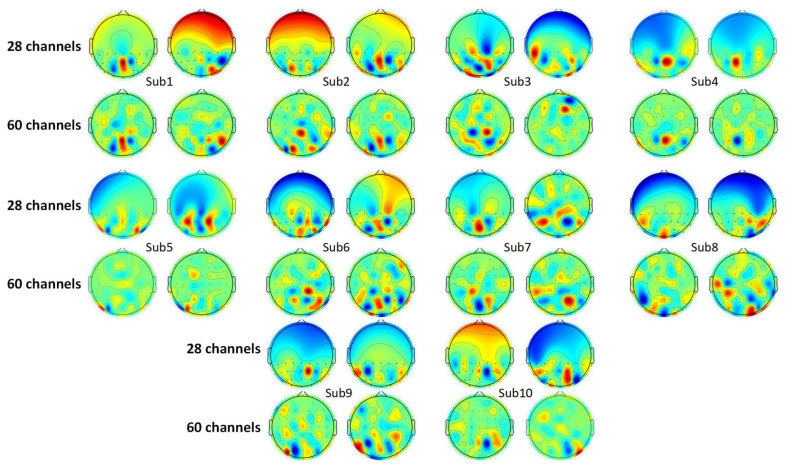
Topological maps of CSP filters.

As introduced in Section 4.1.2, a spatial filter is obtained in the process of CSP feature extraction. This spatial filter consists of a group of weight vectors. The dimension of these vectors matches the number of electrodes. Here, the active degree of each electrode could be represented by the corresponding weight in the vector [58]. Some researchers selected channels of interest according to the weights [59,60]. CSP features obtained with the first vector and the last vector are usually the most distinctive [55]. Thus, the topological graphs of the first and the last weight vectors are selected to analyze the SVI EEG pattern, which is shown in Figure 5. The deep red part and deep blue part denote high positive and negative values of weights. The green part denotes weight, which is close to zero. For each subject, the left maps show the electrodes of interest in the left motion imagination task, and vice versa. Additionally, the topological graphs of 60 electrodes are also given as a contrast to eliminate the influence of electrodes in other regions.

There is little difference between the CSP weight distribution in the 28-electrode map and the 60-electrode map. High weights (points in deep red or deep blue) appear in the occipital lobe and parietal lobe for almost all subjects. However, these regions of interest vary with subjects, and no certain electrode of interest is obtained according to the distribution of CSP weights. But it is evident that most high weights appear in the intersection of the occipital lobe, parietal lobe, and temporal lobe (PO5, PO6, PO7, PO8, P7, and P8 electrodes), and the parietal lobe (PZ, P1, P2, P3, P4, CPZ, CP1, CP2, CP3, and CP4). This intersection corresponds to the V5/MT region [61]. Thus, it is shown that some special EEG patterns may exist in the visuospatial pathway regions.

#### 4.2.2. Analysis of Cross-Correlation Features

**Figure 6:**
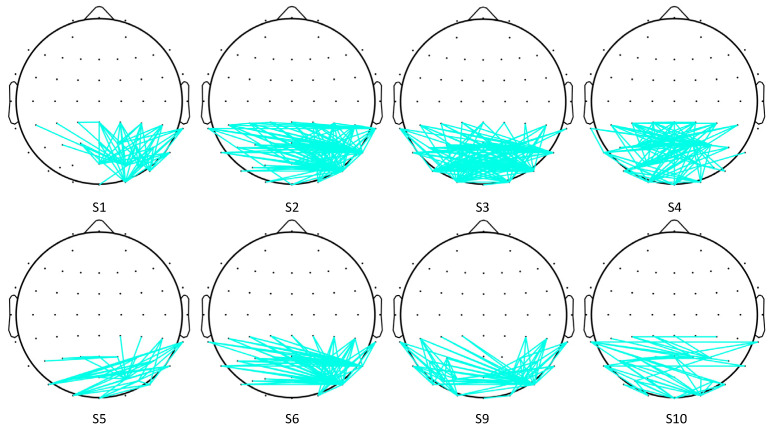
Topographical maps of significant cross-correlation edges.

A *t*-test was applied to observe the difference in cross-correlation features between the two SVI cases. Firstly, the *t*-test results (h = 1 or 0) of each pair of electrodes were determined. Then, only those with significant connectivity edges were retained (h = 1, *p* < 0.01). Here, the remaining edges were defined as significant edges. Significant differences were observed in the cross-correlation networks of eight subjects. The topographical maps of edges with significant differences are shown in Figure 6, where only significant edges are shown. To evaluate which area is the most distinctive in these two SVI tasks, the significant node degree of each electrode was obtained by calculating the number of significant edges of each electrode.

**Figure 7:**
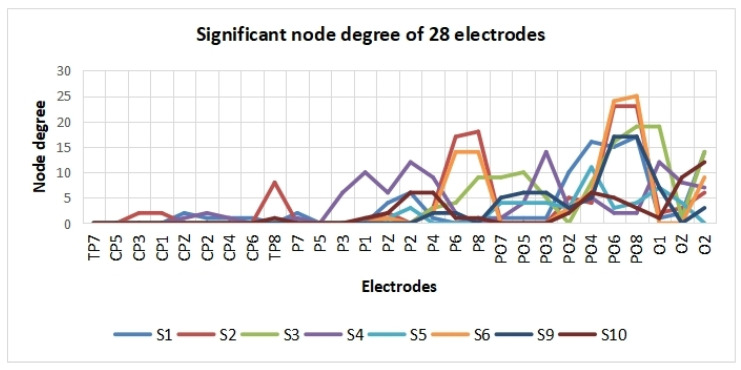
Significant node degrees of electrodes.

According to Figure 6 and Figure 7, the significant edges mainly involve the linkage of PO8 and parietal lobes except for subject 4, subject 5, and subject 10. The right middle temporal lobe (V5/MT in the right hemisphere) is covered by the PO8 electrode, which indicates that cross-correlation features between the visual motion area and parietal lobes are significantly different in the two tasks. This result, to some extent, verifies our hypothesis.

To show the difference in cross-correlation features obtained in the region of interest between the two tasks, the summation of significant edge values between PO8 and parietal electrodes including (PZ, P1-P6, CPZ, and CP1–CP6) was conducted. Before analyzing the edges with a *t*-test, the edge values were normalized to [0, 1] using the min–max normalization method. Then, the insignificant edges were discarded with a *t*-test. Finally, the summation of significant edges between PO8 and the parietal lobe was obtained. The summation values of subjects vary due to different numbers of significant edges for different individuals.

**Figure 8:**
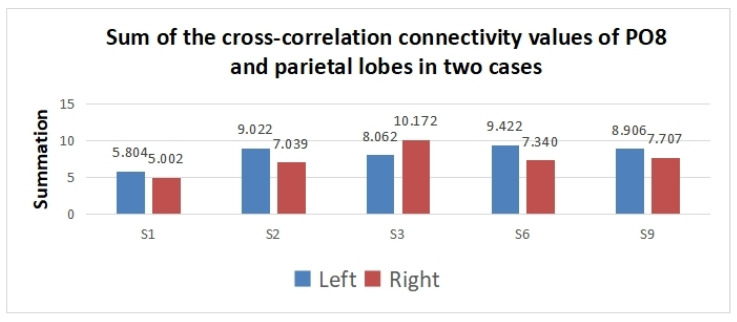
Summation of the significant edge values of PO8 and parietal lobes in two cases.

According to Figure 8, the summation of the PO8-parietal cross-correlation connectivity values is higher in the left SVI case for subjects 1, 2, 6, and 9. However, the opposite results are obtained for subject 3. The result implies that the cross-correlation feature has great classification potential for left/right SVI.

#### 4.2.3. Analysis of Coherence Features

**Figure 9:**
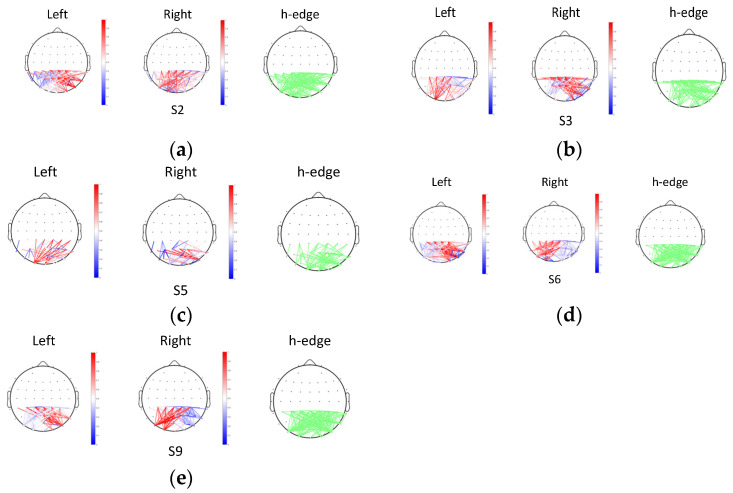
Topographic maps of effective coherence and *t*-test results: (**a**) results of subject 2; (**b**) results of subject 3; (**c**) results of subject 5; (**d**) results of subject 6; and (**e**) results of subject 9.

The topographic maps of coherence FC and the *t*-test results are shown in Figure 9. The topographic maps with a green edge (h-edge) present connectivity values that are significantly different in the two SVI tasks (h = 1, *p* < 0.01). The other two topographic maps present the coherence values of signals in two cases. A deep red edge indicates a high positive connectivity value and a deep blue edge indicates a high negative connectivity value. Here, only the edges with significant differences between the two tasks are retained. Effective coherence features of five subjects are obtained in this work.

According to the h-edge maps, most linkages between the lateral occipital lobes and parietal lobes are significantly different. However, no uniform result is obtained because the coherence distributions of different individuals vary. For subjects 2, 6, and 9, their coherence connectivity values of the right lateral occipital lobes (P8, PO8) and parietal lobes (CPZ, CP1, CP2, CP3, CP4) are stronger in the left SVI task. The connectivity values of the left lateral occipital lobes (PO7) and parietal lobes are stronger in the right SVI task. However, the opposite results are observed for subject 3 and subject 5. 

Previous studies have pointed out that the MT/V5 area provides the strongest sensory signal in response to changes in the direction of translational motion, and the right inferior parietal lobe (rIPL) may be involved in the processing of signals related to orienting of attention [62]. It was found that the right V5/MT (middle temporal) area and the rIPL were activated when a subject perceived the left hemifield stimulation. For the right hemifield stimulation, the left V5/MT area and the rIPL were activated. Our statistical result indicates that different coherence distributions exist in the dorsal pathway area. Although previous work and our work have no direct relationship, both indicate that electrophysiological properties are different in the dorsal pathway area when people perceive different direction information. Using the coherence feature collected from visual motion-related lobes and parietal lobes, it is feasible to discriminate the intention of some subjects, which also supports our hypothesis.

## 5. Spatial Feature-Based Discrimination Model for SVI

### 5.1. Structure of Discrimination Model

In this work, three spatial features of SVI EEG are tested, and a neural network-based single-trial discrimination model is proposed. Considering these three features have different structures, a multi-feature fusion model (MFFM) is designed to satisfy the multiple feature inputs.

**Figure 10:**
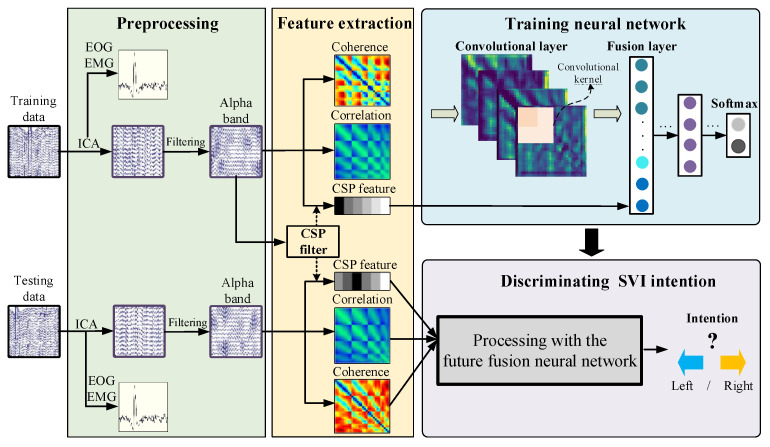
The framework of the multi-feature fusion-based discrimination model for SVI.

The model training process and model testing process are shown in Figure 10. The training process is shown in the upper part and the testing process is shown in the lower part. The coherence features and cross-correlation features are calculated directly with the processed data. When calculating CSP features, a CSP filter is trained first with the training data. Then, the CSP features for training and testing are extracted with the CSP filter. Finally, a neural network for SVI discrimination is trained with the extracted features in the training process and evaluated in the testing process. This model uses the original EEG data as input and decodes the users’ SVI intention (imagine motion in left or right).

### 5.2. Selective Kernel Network (SKN)-Based SVI Discrimination Model

One key issue for single-trial discrimination in this study is the heterogeneity in the multi-feature inputs. As mentioned in Section 4.1, the shape of the functional connectivity features is 28 × 28, and the CSP feature is a 1-D feature with the shape of 1 × 6. It is not reasonable to combine these features directly. Therefore, it is necessary to adjust the dimensions of features without losing useful information. A neural network (NN) is an effective model for multi-heterogenous inputs. Useful information can be extracted with convolution operation from high-dimensional features. Dimension reduction can be achieved with the forward propagation mechanism. When processed with networks, heterogeneous features are transformed into some abstract features with reduced heterogeneity. Therefore, a convolutional neural network with parallel inputs is built to realize proper feature fusion.

**Figure 11:**
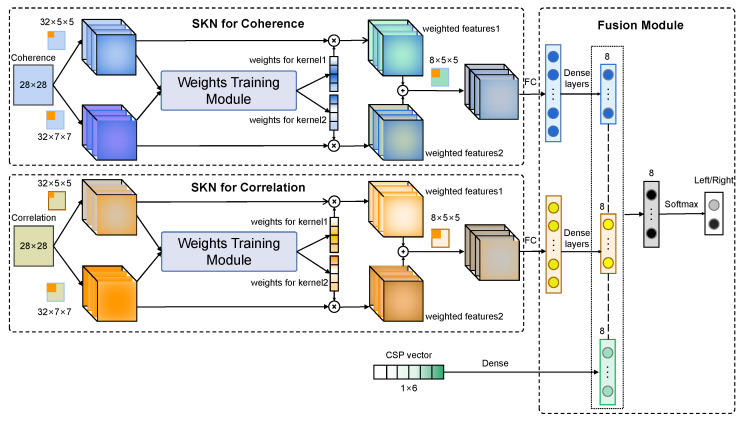
Structure of the multi-feature fusion model (MFFM).

Another problem is that effective edge features of functional connectivity maps are difficult to detect because they are small and discrete in FC maps. In the CNN model, different information can be obtained with kernels of different sizes. Considering the complicated distribution of edges in FC maps, it is necessary to capture more information under multiple receptive fields to obtain affluent features to improve classification accuracy. Furthermore, the weights of multi-scale features are adaptively adjusted to strengthen the effective features and diminish the redundant ones. The selective kernel network (SKN) is a novel multi-scale attention mechanism that can be used to learn the adaptive weights for convolutional layers with different kernels [63]. In this work, the SKN module is embedded in the CNN structure with multiple inputs to obtain multi-scale features and assign adaptive weights to these features. The structure of the MFFM is shown in Figure 11.

Three features including coherence, cross-correlation, and CSP are the inputs of the model. This model outputs the intention of imagination (motion in left/right) ultimately. There are three modules in this structure, including the SKN module for coherence features, the SKN module for cross-correlation features, and a feature fusion module. In the first module, a 32 × 5 × 5 kernel set and a 32 × 7 × 7 kernel set are selected to capture the edge information on the coherence feature map. Then, two sets of features in the first layer are fed into the weight training module to obtain the adaptive weights for each kernel. Two group features are weighted by multiplying them with the weights in the channel dimension. Finally, these two feature sets are added, and the accumulated feature is processed with an 8 × 5 × 5 kernel set. The SKN structure for cross-correlation features is the same as that of the coherence features. All processed 2-D deep features are expanded in the Full-Connect (FC) layer. However, it is still unreasonable to make a feature combination directly due to the huge difference between the dimension of the CSP feature and that of the expanded FC feature. Therefore, the dimension of expanded FC features extracted with the CNN is reduced using several dense layers. Three dense layers are applied in the model. The numbers of neurons in the three layers are 1024, 256, and 8, respectively. A hidden layer with eight elements is applied for the CSP features. In the fusion module, three deep features are concatenated directly. Then, a hidden layer with eight neurons and a softmax layer with two neurons are set to obtain the final result.

**Figure 12:**
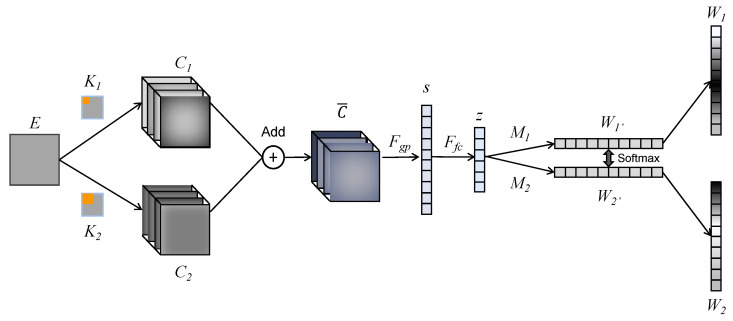
Structure of SKN.

The expanded structure of SKN is shown in Figure 12. Here, matrix *E* with the shape of 28 × 28 denotes the FC input (coherence map or cross-correlation map). Then, the *K*_1_ (32 × 5 × 5) and *K*_2_ (32 × 7 × 7) kernel sets are applied in the convolutional layer to obtain the *C*_1_ and *C*_2_ feature sets. Then, *C*_1_ and *C*_2_ are added using an element-wise summation to obtain the mixing feature set (32 × 28 × 28).

(7) $$\overset{-}{C}=C_{1}+C_{2}$$

Then, a global average pooling layer is used to obtain a 1 × 32 vector *s*, which contains the channel information of the mixed feature. Vector *s* is calculated with Formula (8). Here, both *H* and *W* are 28, which denotes the spatial dimension of the feature in each channel.

(8) $$s=F_{gp}(\overset{-}{C})=\frac{\sum_{j=1}^{H}\sum_{i=1}^{W}\overset{-}{C}(i,j)}{H\times W}$$

Then, *s* is converted to *z* with Formula (9), where *W* denotes a mapping matrix with the shape of 32 × 32, *β* denotes the batch normalization, and *δ* denotes the ReLU function. The dimension of *z* is selected as 32, referring to the previous work.

(9) $$z=F_{fc}(s)=\delta (\beta (Ws))$$

*z* is multiplied with mapping matrix *M*_1_ and *M*_2_ and then converted into two weight vectors *W*_1_′ and *W*_2_′. The dimensions of *W*_1_′ and *W*_2_′ are the same as the number of feature channels. Then, a softmax mechanism is applied on *W*_1_′ and *W*_2_′ to obtain the ultimate weight vectors *W*_1_ and *W*_2_. Here, *w*_1*c*_ and *w*_2*c*_ denote the *c*-th element of *W*_1_ and *W*_2_, and *m*_1*c*_ and *m*_2*c*_ denote the *c*-th vector of *M*_1_ and *M*_2_. The softmax layer is shown in Formula (10).

(10) $$w_{2c}=\frac{e^{m_{2c}z}}{e^{m_{1c}z}+e^{m_{2c}z}}$$

(11) $$w_{1c}+w_{2c}=1$$

### 5.3. Data Processing

In this work, data sets of 10 subjects are available. Each data set consists of the left SVI EEG and the right SVI EEG. The training set accounts for 75%, and the testing set accounts for 25%. A four-fold cross-validation was used to evaluate the discrimination performance of spatial features of SVI EEG.

The parameters of the model implementation are shown in Table 4. Here, two dropout layers are set at the fully connected layers to prevent overfitting. Feature extraction was implemented with Matlab2020. Model training and testing were implemented with the Keras toolkit in Python 3.7.

The purpose of this study is to prove whether the spatial features of SVI EEG are distinctive and construct a feasible SVI discrimination model to realize good classification. The multi-feature fusion model (MFFM) was trained with the three features mentioned in Section 4.1. To evaluate the performance of a single spatial feature, the single-input model was also tested in this work. In particular, an SVM classifier was selected for single CSP features due to their low dimensionality. In addition to the spatial features, some other features were also tested as a comparison. The “Original data (OD)+CNN” method is used to make a comparison between the spatial features and original data. The original data filtered in the alpha band were used as the input of the “Original data (OD)+CNN” method. “PSD+SVM” and “HHT Marginal Spectrum (HHTMS)+SVM” were tested to compare the spatial features with frequency energy features. All the energy features were extracted with data in the alpha band.

### 5.4. Discrimination Performance

The classification results of the different methods are shown in Table 5. The best accuracy of each subject is in bold. No good classification accuracy was obtained when the original data or the frequency energy features were taken as the inputs. The *t*-test was conducted to evaluate whether the classification results of the spatial input methods and the other methods are significantly different. The *t*-test result is given in Table 6. Here, only the difference between the result of “OD+CNN” and “Coherence+SKN” is not significant due to the invalid coherence features of some subjects. In general, the accuracy is significantly higher when the spatial features are used for classification. The best accuracy of each subject is obtained with spatial inputs. The best accuracy in this experiment reaches 0.93. As a result, it is found that the spatial information of SVI EEG is identifiable and more effective for single-trial discrimination compared with the original data and conventional frequency features.

Although good classification accuracy is obtained with spatial SVI features, the multi-input results and single coherence input results of subjects 4, 7, 8, and 10 are not ideal. The correlation value of “Coherence+SKN” accuracy and “Three-input+MFFM” accuracy is 0.95, which means that the poor accuracy of multi-inputs may be mainly caused by the invalid coherence features.

To evaluate the performance of the classifiers, five metrics including accuracy, area under the curve (AUC), precision, recall rate, and the F-measure value are calculated, and the results are shown in Table 7. Here, the classifiers of the best-performing methods are used to calculate the metrics. These classifiers perform well, and all of the average metrics are above 80%, which proves that the discrimination models used in this study are feasible in SVI tasks.

Overall, a good result is obtained by combining these spatial features and the proposed classification model. Compared with conventional frequency features like OD, PSD, and HHTMS, spatial features show good performance and robustness in the single-trial test. This can be attributed to the fact that a subject’s EEG frequency energy (similar to PSD) does not have a significant difference under different SVI tasks. Thus, the classification result is not ideal when using frequency features. According to Section 4.2, most subjects can obtain stable spatial feature patterns. The spatial patterns in the same data set are consistent. For an individual, these spatial patterns are significantly different between classes and change little within classes. This is why spatial features perform well and robustly in the single trial test. However, according to the analysis of features, not all the subjects obtained good spatial features, and no uniform spatial pattern was obtained among all subjects. This means that every time a new user uses SVI, feature calibration is needed, which causes poor transferring performance of the system.

Concerning the proposed MFFM, this model integrates three spatial features reasonably with a deep learning structure. According to Table 7, MFFM performs better than single-feature-based classification (CSP+SVM), as the complementary property of three features is fully utilized by fusion. However, the deep learning model is prone to overfitting problems because of its large parameter structure. Once the covariance in the feature is high or there are not enough samples, MFFM may perform even worse than the simple classifier.

## 6. Conclusions

In this work, we studied an SVI-based EEG discrimination method for CAD manipulations. This paradigm can be used to realize 1-D translation manipulation, which allows designers to send their commands to the CAD manipulation system mentally and intuitively. A screw assembly experiment was conducted, and three types of spatial features including CSP features, cross-correlation peak-based FC, and coherence-based FC were analyzed. The statistical results verified our hypothesis that features extracted in the visuospatial perception area can be different in various SVI cases. Finally, feasible classification models were built for these EEG features. The method proposed provides theoretical support for discriminating the intention of CAD object translation with good classification performance.

The EEG-based interaction mode proposed in this work can be used to output designers’ intention of a single translation in the CAD environment. In this way, we could build a direct “end to end” interaction between CAD and the brain to overcome the redundant operations of other models and make designers express intentions more intuitively. Moreover, the SVI paradigm is more intuitional and natural than convenient BCI paradigms, and we verified the feasibility of spatial features and built a classification model that performs well. Thus, our findings provided some theoretical support for subsequent research on BCI improvement. 

However, some limitations still exist in our work. Firstly, the sample size of subjects in the experiment is relatively small. Although this data size is enough to support our findings, we need to recruit more subjects (especially females) and collect more data sets to make our model more robust. Secondly, EEG is a non-stationary signal, which varies from individual to individual. In addition to increasing the number of subjects, we also need to explore more features in the future as a supplement to improve the discrimination results. 

In the future, we will build an online system based on the findings of this work and evaluate its performance. To put our findings into real-world CAD applications, our next challenge is to develop an asynchronous BCI [64] into which our feature extraction methods and the trained model are embedded. We also want to connect the online BCI system to CAD with a high ITR (Information Translate Rate). Moreover, as an important factor in interaction, the user experience cannot be ignored. We will record some indicators like blood oxygenation and task complement time to evaluate the long-term usability of the practical application. Furthermore, only the SVI of translation manipulation is studied in this work, while real-world CAD requires more commands. Thus, some other CAD manipulation functions like rotation and zooming are expected to be realized with EEG recognition in our future work.

## Figures and Tables

**Table 1:** VVIQ scores of ten subjects.

ID	1	2	3	4	5	6	7	8	9	10
Score	58	72	66	70	59	68	54	58	70	56

**Table 2:** Imagination strategies of different subjects.

ID	Strategy
1	Imagine the movement of the screw itself
2	Imagine the movement of the screw itself
3	Imagine big arrows following the screw
4	Imagine the movement of the screw itself
5	Imagine the movement of the screw itself
6	Imagine an extending strap following the screw
7	Imagine the movement of the screw itself
8	Imagine a series of arrows following the screw
9	Imagine the movement of the screw itself
10	Imagine the movement of the screw itself

**Table 3:** The *t*-test result of CSP features.

ID	f1	f2	f3	f4	f5	f6
1	*p* = 2.6 × 10^−8^ h = 1	*p* = 5.5 × 10^−1^ h = 0	*p* = 7.0 × 10^−1^ h = 0	*p* = 1.8 × 10^−1^ h = 0	*p* = 3.5 × 10^−5^ h = 1	*p* = 3.4 × 10^−12^ h = 1
2	*p* = 8.4 × 10^−12^ h = 1	*p* = 1.4 × 10^−3^ h = 1	*p* = 4.0 × 10^−2^ h = 1	*p* = 5.8 × 10^−4^ h = 1	*p* = 8.6 × 10^−4^ h = 1	*p* = 9.2 × 10^−6^ h = 1
3	*p* = 1.6 × 10^−1^ h = 0	*p* = 2.5 × 10^−3^ h = 1	*p* = 2.5 × 10^−3^ h = 1	*p* = 9.6 × 10^−2^ h = 0	*p* = 2.1 × 10^−3^ h = 1	*p* = 5.2 × 10^−4^ h = 1
4	*p* = 2.3 × 10^−4^ h = 1	*p* = 2.9 × 10^−2^ h = 1	*p* = 1.7 × 10^−2^ h = 1	*p* = 6.5 × 10^−1^ h = 0	*p* = 1.7 × 10^−1^ h = 0	*p* = 1.2 × 10^−5^ h = 1
5	*p* = 1.3 × 10^−4^ h = 1	*p* = 4.3 × 10^−6^ h = 1	*p* = 1.4 × 10^−1^ h = 0	*p* = 5.3 × 10^−1^ h = 0	*p* = 1.8 × 10^−3^ h = 1	*p* = 2.3 × 10^−4^ h = 1
6	*p* = 2.0 × 10^−5^ h = 1	*p* = 9.8 × 10^−5^ h = 1	*p* = 5.2 × 10^−1^ h = 0	*p* = 1.8 × 10^−1^ h = 0	*p* = 9.4 × 10^−3^ h = 1	*p* = 1.3 × 10^−5^ h = 1
7	*p* = 4.9 × 10^−4^ h = 1	*p* = 6.2 × 10^−6^ h = 1	*p* = 3.8 × 10^−2^ h = 1	*p* = 3.7 × 10^−2^ h = 1	*p* = 5.5 × 10^−3^ h = 1	*p* = 1.7 × 10^−2^ h = 1
8	*p* = 4.4 × 10^−3^ h = 1	*p* = 1.1 × 10^−1^ h = 0	*p* = 1.5 × 10^−3^ h = 1	*p* = 4.5 × 10^−2^ h = 1	*p* = 1.9 × 10^−1^ h = 0	*p* = 8.0 × 10^−8^ h = 1
9	*p* = 1.3 × 10^−6^ h = 1	*p* = 4.5 × 10^−3^ h = 1	*p* = 3.4 × 10^−1^ h = 0	*p* = 9.3 × 10^−1^ h = 0	*p* = 3.1 × 10^−4^ h = 1	*p* = 1.1 × 10^−5^ h = 1
10	*p* = 5.1 × 10^−4^ h = 1	*p* = 9.9 × 10^−2^ h = 0	*p* = 1.5 × 10^−1^ h = 0	*p* = 7.1 × 10^−1^ h = 0	*p* = 4.5 × 10^−4^ h = 1	*p* = 9.5 × 10^−4^ h = 1

**Table 4:** Parameters of model implementation.

Parameter	Value
Ratio of training set to testing set	3:1
Optimizer	Adam
Loss function	Binary crossentropy
Learning rate	5 × 10^−4^
Dropout value	0.2
Training epochs	400

**Table 5:** Classification accuracy of different methods.

ID	OD+CNN	PSD+SVM	HHTMS +SVM	CSP+SVM	Cross-Correlation +SKN	Coherence+SKN	Three Inputs+ MFFM
1	0.70	0.68	0.52	0.85	0.84	0.75	**0.87**
2	0.75	0.72	0.62	0.87	0.87	0.84	**0.93**
3	0.76	0.68	0.65	0.75	0.78	0.84	**0.86**
4	0.68	0.73	0.66	**0.87**	0.74	0.65	0.75
5	0.60	0.70	0.65	0.76	0.75	0.78	**0.83**
6	0.70	0.70	0.68	0.81	0.82	0.88	**0.92**
7	0.72	0.67	0.66	**0.79**	0.72	0.59	0.63
8	0.68	0.61	0.60	**0.82**	0.71	0.66	0.73
9	0.80	0.69	0.62	0.84	0.83	0.83	**0.88**
10	0.63	0.55	0.58	**0.80**	0.78	0.68	0.76
Average	0.70	0.67	0.62	0.82	0.78	0.75	0.82

**Table 6:** *p*-values of the *t*-test of different methods (α = 0.05).

Method	CSP+SVM	Cross-Correlation+SKN	Coherence+SKN	Multi-Input +MFFM
OD+CNN	1.08 × 10^−4^	1.30 × 10^−3^	0.17	4.90 × 10^−3^
PSD+SVM	3.57 × 10^−6^	2.13 × 10^−5^	3.93 × 10^−2^	6.45 × 10^−4^
HHTMS+SVM	2.00 × 10^−8^	3.14 × 10^−8^	1.90 × 10^−3^	2.20 × 10^−5^

**Table 7:** Evaluation metrics of the classifiers with the best performance.

ID	Method	Accuracy	AUC	Precision	Recall	F-Measure
1	MFFM	0.87	0.87	0.85	0.85	0.85
2	MFFM	0.93	0.93	0.91	0.91	0.91
3	MFFM	0.86	0.84	0.83	0.83	0.83
4	CSP+SVM	0.87	0.86	0.85	0.85	0.85
5	MFFM	0.83	0.83	0.82	0.82	0.82
6	MFFM	0.92	0.92	0.90	0.90	0.90
7	CSP+SVM	0.79	0.79	0.78	0.78	0.78
8	CSP+SVM	0.82	0.80	0.81	0.81	0.80
9	MFFM	0.88	0.88	0.87	0.87	0.87
10	CSP+SVM	0.80	0.79	0.78	0.78	0.78
Average	--	0.86	0.85	0.84	0.84	0.84

## Data Availability

In the informed consent form filled out by the subjects before the start of the experiment, it was written “Only the research team will have access to the data”. In order to protect the privacy of the subjects, we decided not to disclose the experimental data.
